# Mummified Oligocene fruits of *Schima* (Theaceae) and their systematic and biogeographic implications

**DOI:** 10.1038/s41598-017-04349-6

**Published:** 2017-06-21

**Authors:** Xiang-Gang Shi, Qiong-Yao Fu, Jian-Hua Jin, Cheng Quan

**Affiliations:** 1 State Key Laboratory of Biocontrol and Guangdong Provincial Key Laboratory of Plant Resources, School of Life Sciences, Sun Yat-sen University, Guangzhou, 510275 China; 2 Research Center of Palaeontology & Stratigraphy, and MOE Key-Lab for Evolution of Past Life and Environment in Northeast Asia, Jilin University, Changchun, 130026 China

## Abstract

The genus *Schima* includes about 20 species and is distributed only in southern China and adjacent areas of Asia. The previous molecular phylogenetic analysis suggested *Schima* is in the tribe *Gordoniae*, along with *Gordonia* and *Franklinia*. However, because few fossils have been reported, the biogeographic origin of *Schima* is still poorly known. In this paper mummified fossil fruits of *Schima* are described from the upper Oligocene Yongning Formation of the Nanning Basin, Guangxi, South China. In gross morphology, the new fossil species, *Schima kwangsiensis*, is similar to the extant *S. superba* by its pentacarpellate, loculicidally dehiscent capsules, 5 imbricate sepals, pedicels with bracteoles and marginally winged seeds. Due to its excellent preservation, the new species may provide sufficient details for understanding the early evolutionary and phytogeographic history of the genus. Morphological clustering analysis shows that the new fossil species is closely related to two extant species (*S*. *wallichii* and *S*. *superba*) in the genus, implying that they may belong to an ancient taxon that occurs earlier than the others. More importantly, this discovery represents the earliest record of this genus in Asia and it explicitly moves the fossil record back to the late Oligocene in this region.

## Introduction

**Figure 1:**
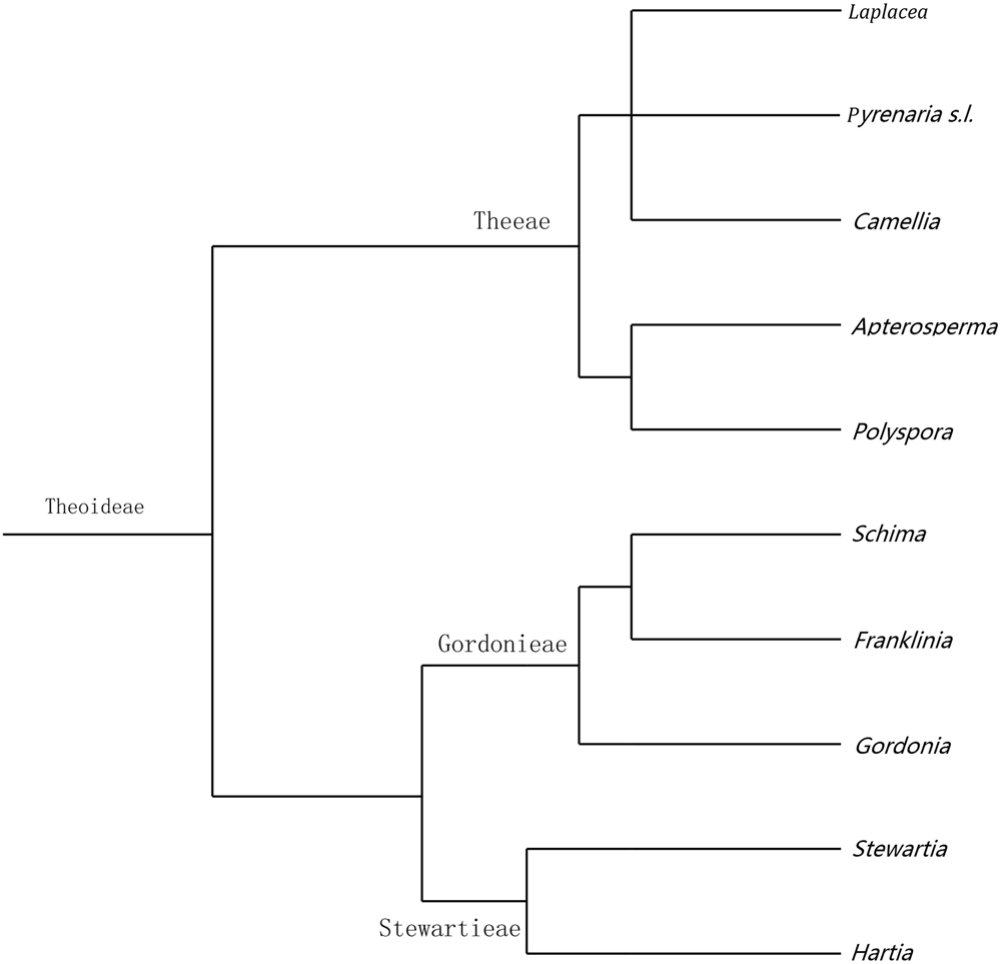
A simplified phylogenetic tree of the Theoideae, drawn by X. G. Shi according the references^10–13^ herein using Adobe Photoshop CS5 (Adobe Inc., San Jose, California, USA).

*Schima*, a genus within the Theaceae in Asia, inhabits subtropical to tropical regions across southern and southeastern Asia, from the eastern Himalaya of Nepal and eastern India across Indochina, southern China, Taiwan, and the Ryukyu Islands^1^. There are about 20 species, including six species endemic to China^2, 3^. It is mainly characterized by marginally winged seeds and capsules with persistent columella. Traditionally, the genus belongs to the subfamily Theoideae and is usually placed in the tribe *Gordoniae* with *Gordonia, Franklinia* and *Laplacea* ^4–6^, or in the tribe *Schimeae* with *Franklinia* and *Apterasperma* ^7–9^. Recent molecular phylogenetic analysis showed *Schima* is closely related to *Gordonia* and *Franklinia*, forming the monophyletic tribe of *Gordoniae* ^10–13^ (Fig. 1).

**Figure 2:**
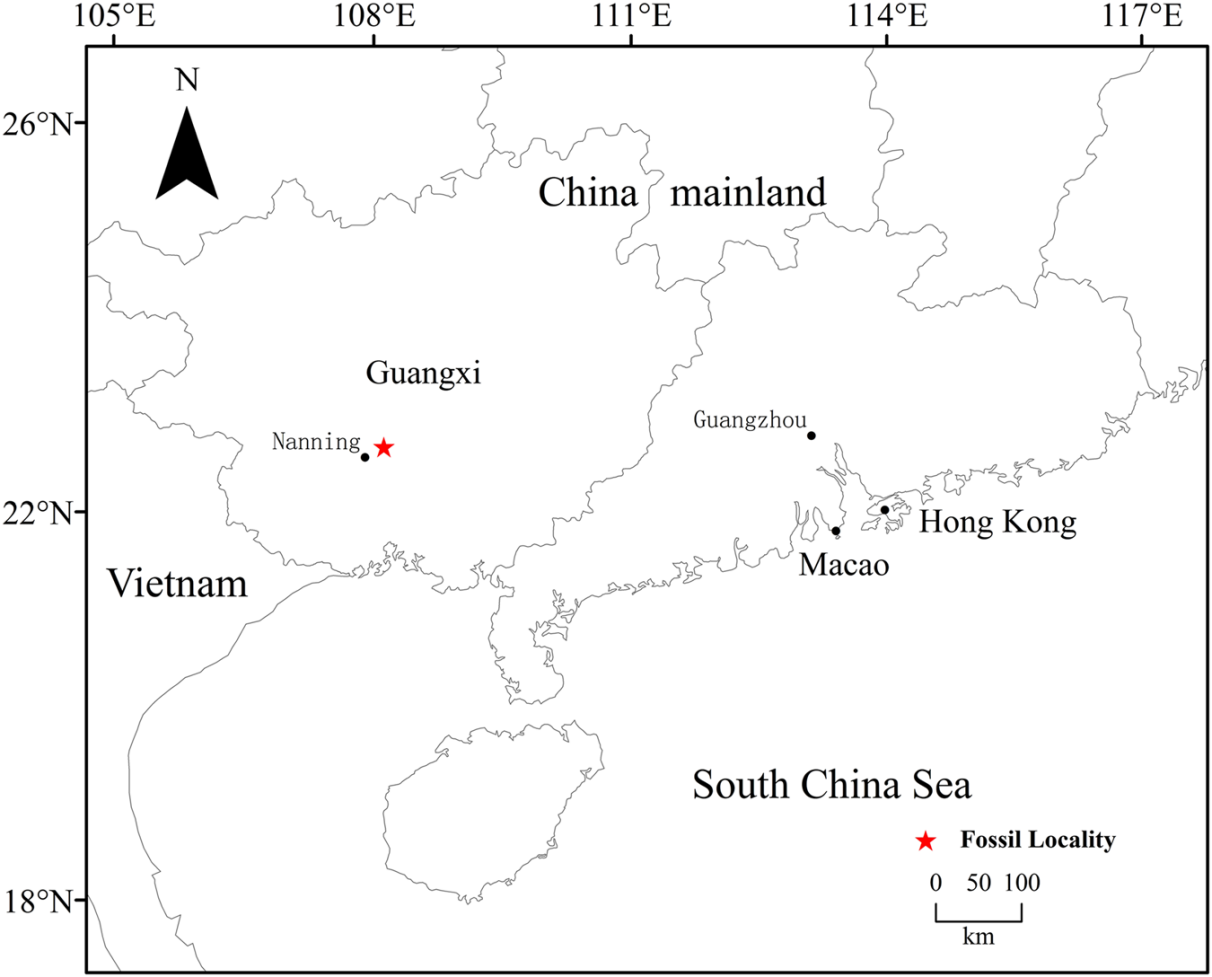
Geographic map of the Nanning Basin, Guangxi Province, generated by X. G. Shi, using Arcgis 9.3 (ESRI, Redlands, CA, USA).

Fruit characters are considered to be of crucial systematic importance in the modern Theaceae^8^, but fossil fruits of *Schima* are very rare^14, 15^. Here, we describe numerous mummified *Schima* fruits collected from a plant fossil Konservat Lagerstätte reported recently^16^ in the late Oligocene Yongning Formation of Nanning Basin of Guangxi Province, South China (22°52′50′′ N, 108°25′2′′E, Fig. 2).

The Nanning Basin is located in Nanning City, Guangxi Province and it mainly consists of three formations: the Ducun Formation, the Yongjiang Formation and the Yongning Formation^17^. According to their respective lithic facies, the Yongning Formation is subdivided into the upper, middle and lower parts^16^. Of these, the upper part is rich in mummified fossils, and is mainly composed of bluish gray clayey mudstone, interlined with a few coal seams and thin sandstones. Based on uncovered mammal fossils of *Anthracotherium chanlingensis* Zhao, *Anthracokeryx kwangsiensis* Qiu, and *Heothema* sp. from Changlin, Xiaoji, and Guzang in the same basin, the age of the upper part is determined as the late Oligocene^16, 17^. Therefore, this occurrence is the earliest definite record of the genus *Schima* in Asia and it moves explicitly the fossil record back to the late Oligocene in Asia. In addition, the new finding with exceptional preservation is the first fossil fruit with a pedicel in the genus. The purpose of this paper is to evaluate the new fossil record of *Schima* and further discuss systematics and phytogeographical history of the genus based on integrated evidence from megafossils and morphological clustering analysis.

## Results

Systematics

**Class** Magnoliopsida Brongn.

**Order** Ericales Bercht. & J. Presl.

**Family** Theaceae Mirbel.

**Genus** *Schima* Reinwardt ex Blume.

**Species** *Schima kwangsiensis* X. G. Shi, C. Quan et J. H. Jin sp. nov.

### Holotype

**Figure 3:**
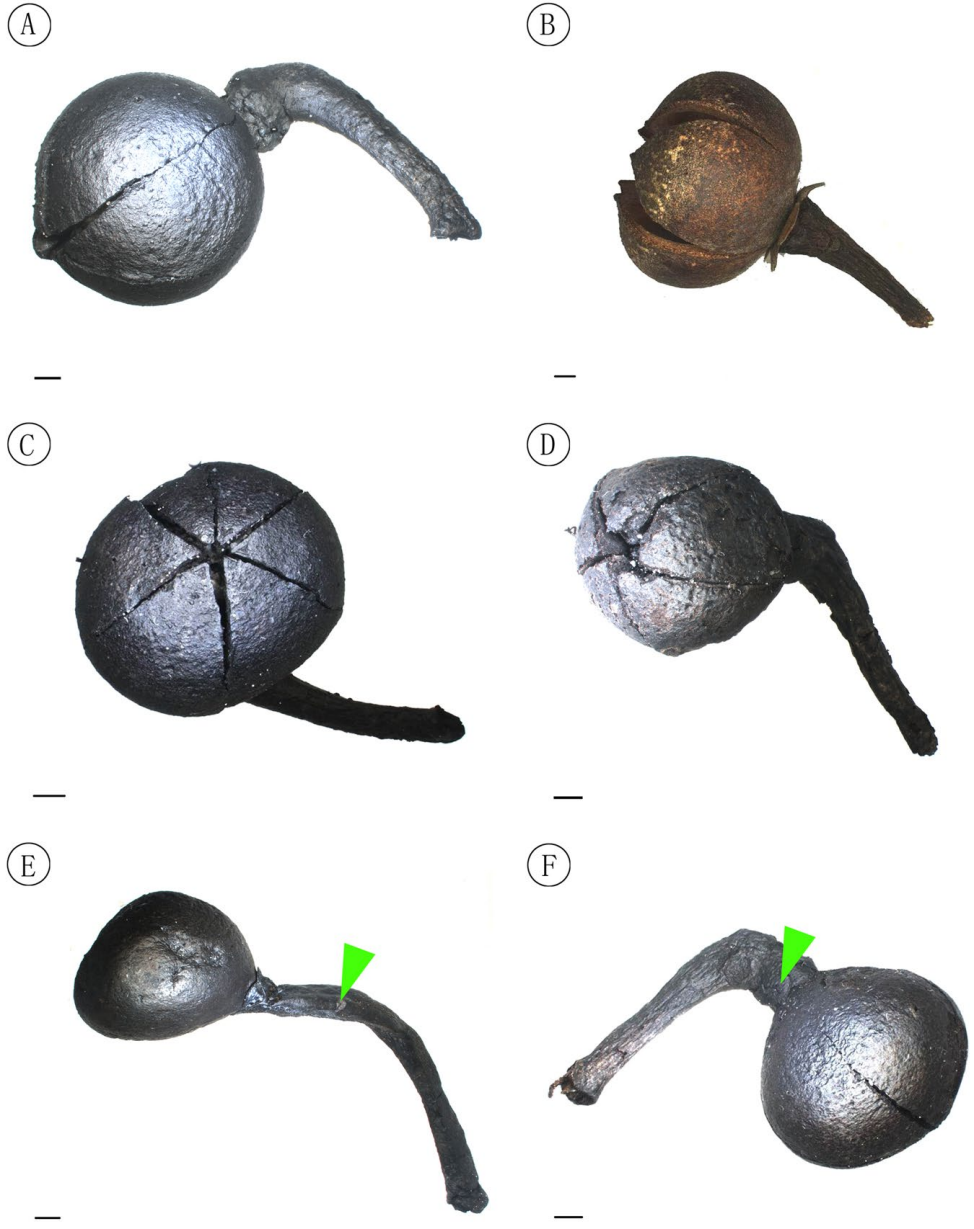
Fruits with pedicels of *Schima kwangsiensis* sp. nov. and extant *Schima superba*. (**A**) The fossil fruit showing the globose capsule, and the apically thickened and recurved pedicel (holotype, specimen number NNF-070). (**B**) A modern fruit of the species *Schima supberba*, collected from the Heishiding Nature Reserve, Guangdong Province. (**C**) The top surface of A showing six loculicidally dehiscent capsule. (**D**) Five loculicidally dehiscent capsules, paratype, specimen number NNF-074. (**E**) A capsule and its long pedicel with bracteole scar (green arrowhead), paratype, specimen number NNF-075. (**F**) Dehiscent capsule showing semiorbicular sepals (green arrowhead), paratype, specimen number NNF-073. Scale bar = 2 mm. Processed and drawn by X. G. Shi using Adobe Photoshop CS5.

NNF-070 (Fig. 3A,C) (designated here).

### Paratypes

**Figure 4:**
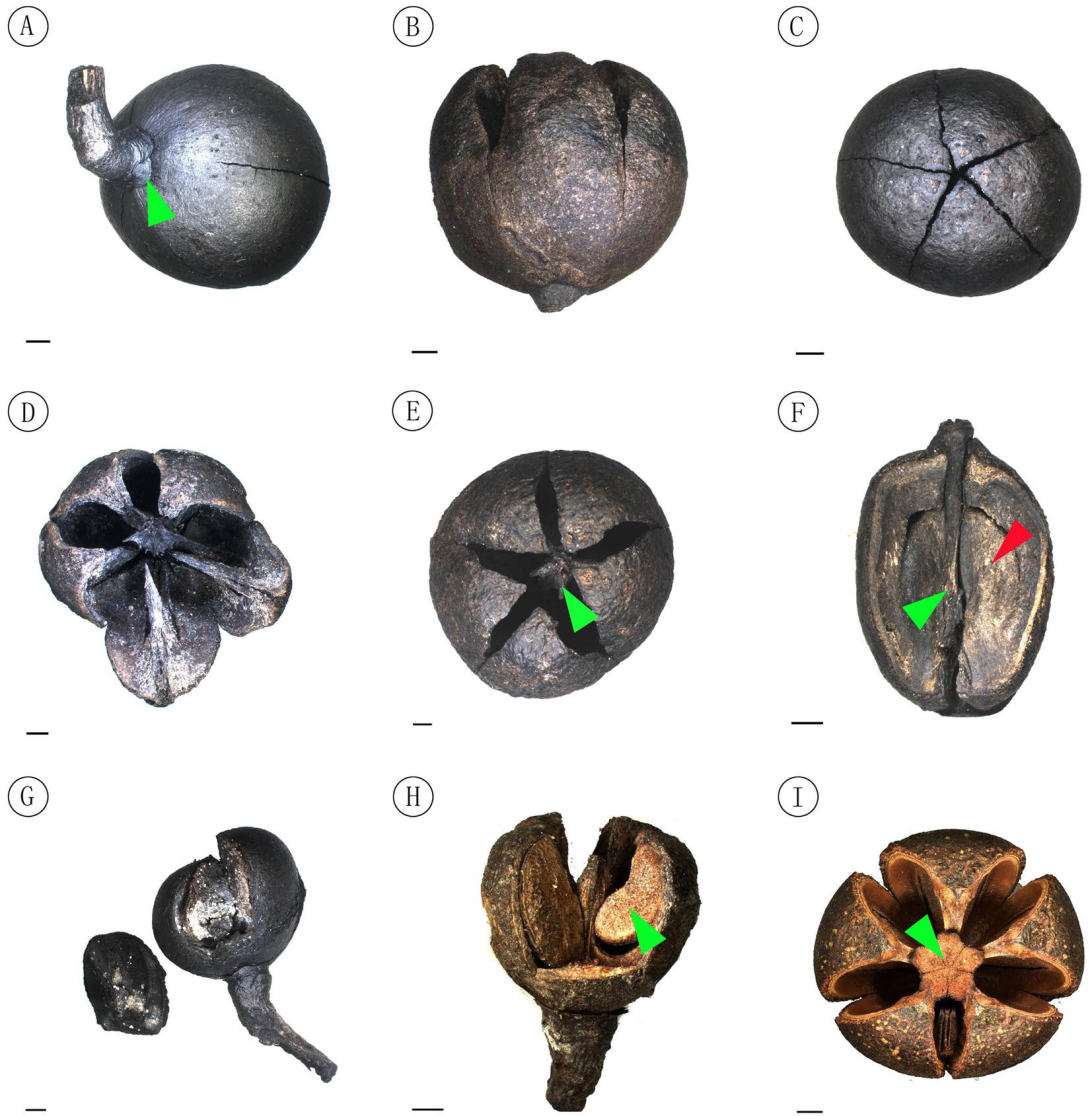
Fruit structure of *Schima kwangsiensis* sp. nov. and extant *Schima superba*. (**A–G**) Fossil fruits: (**A**) Dehiscent capsule showing semiorbicular sepals (green arrowhead), specimen number NNF-072. (**B**) Dehiscent capsule showing splits running down the upper 2/3 of the capsule length, specimen number NNF-1493. (**C**) Dehiscent capsule with five ventral sutures, specimen number NNF-1495. **(D)** Dehiscent capsule showing 5 valves and dissepiments, specimen number NNF-1490. **(E)** Dehiscent capsule showing five locules and the persistent five-angled columella (green arrowhead), specimen number NNF-1491. **(F)** Half capsule showing the persistent columella (green arrowhead) and reniform seed (red arrowhead), specimen number NNF-1499. (**G**) Dehiscent capsule with a deciduous valve, specimen number NNF-076. **(H,I)** Modern fruits of the species *Schima supberba*: (**H**) Partially dehiscent capsule showing reniform seeds. **(I)** Pentacarpellate, loculicidally dehiscent capsule showing apically 5-angled columella (green arrowhead). Scale bar = 2 mm. Processed and drawn by X. G. Shi using Adobe Photoshop CS5.

NNF-072 (Fig. 4A); NNF-073 (Fig. 3F); NNF-074 (Fig. 3D); NNF-075 (Fig. 3E); NNF-076 (Fig. 4G); NNF-1490 (Fig. 4D); NNF-1491 (Fig. 4E); NNF-1493 (Fig. 4B); NNF-1495 (Fig. 4C); NNF-1499 (Fig. 4F) (designated here).

### Locality

Santang Town of Nanning City, Guangxi Province, South China.

### Stratigraphic Horizon

Yongning Formation, late Oligocene.

### Repository

The Museum of Biology of Sun Yat-sen University (SYS), Guangzhou, China.

### Etymology

The epithet “*kwangsiensis*” means the fossil is collected in Kwangsi (Guangxi) Province, China.

### Specific Diagnosis

Pedicel 4–5 cm long; bracteoles 2–3; sepals 5, imbricate, persistent, slightly connate at base; capsule globose or depressed globose, pericarp woody, loculicidally splitting into 5–6 valves; columella persistent, stout, extending for 2/3 of locule length, apically 5-angled; seed reniform and flat, with a marginally membranous wing.

## Description

**Figure 5:**
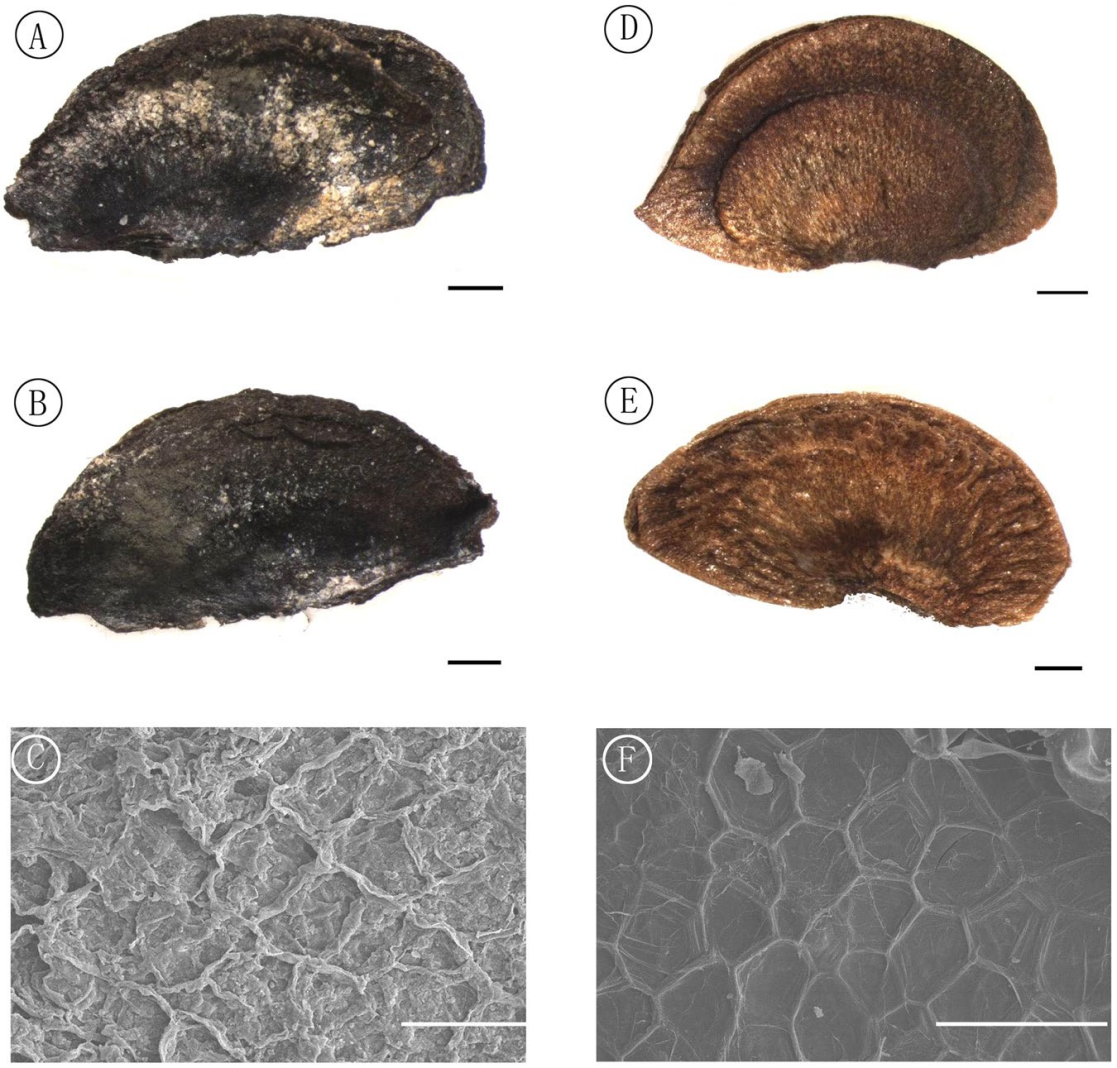
Seed and seed coat structures of *Schima kwangsiensis* sp. nov. and extant *Schima superba*. (**A,B**) The two sides of a reniform fossil seed and its wing, specimen number NNF-076. Scale bar = 1 mm. **(C)** Details of the seed coat structure of A, showing irregular reticulate epidermal ornamentation, Scale bar = 100 μm. **(D,E)** The two sides of a modern seed of the species *Schima superba*, Scale bar = 1 mm. **(F)** Details of the seed coat structure of D, showing regular reticulate epidermal ornamentation, Scale bar = 100 μm. Processed and drawn by X. G. Shi using Adobe Photoshop CS5.

Pedicel slender, apically thickened and recurved, bracteoles 2–3, caduous, away from sepals. Sepals 5, persistent, imbricate, basally slightly connate, semiorbicular, 2–3 mm in diameter. Capsule globose, 2–2.5 cm in diameter, pericarp woody, splitting for 1/2–2/3 length into 5–6 valves (Figs 3A,C–F and 4A–C); columella persistent, stout, extending for 2/3 or more of locule length, apically 5-angled with an enlarged face inside each of the five locules (Fig. 4D–E). Seeds small, reniform, flat, with a clear marginal membranous wings, 7.5–8 mm long, 4–5 mm wide including wing, sub-campylotropous attached to the columella. Hilum linear and short (Figs 4F–G and 5A,B). Seed coat with conspicuous protruding curved ridges that form irregularly reticulate ornamentations (Fig. 5C).

### Comparison

The new fossil species is characterized by its 5-loculed loculicidally dehiscent capsules, 5 imbricate sepals and pedicels with bracteoles. These features are consistent with the subfamily Theoideae of Theaceae and readily distinguish the fossils from fruits of other angiosperm families^18^. Modern Theoideae are divided into two main groups according to seed characters: one is composed of taxa bearing wingless seeds and includes *Camellia*, *Tutcheria*, *Pyrenaria, Parapyrenaria, Apterosperma* and *Franklinia*; the other encompasses taxa with winged seeds such as *Gordonia*, *Laplacea*, *Schima*, *Hartia* and *Stewartia* ^6^. Furthermore, all the genera with winged seeds can be further divided into two groups based on seeds having either apical or marginal wings. Those genera with marginally winged seeds mainly include *Schima*, *Stewartia* and *Hartia*, but the columella in fruit of *Stewartia* and *Hartia* is incomplete or only extends ca. 1/2 the length of the locule, easily distinguishing these genera. As for *Franklinia*, its immature seeds sometimes have diminished wings, but the dehiscence mode of the capsules in *Franklinia* is unique that they have both loculicidal dehiscence and septicidal dehiscence, and the capsule valves rarely adheres to the central part of the columella^8, 9^. Therefore, compared with other related genera, these fossil fruits belong to *Schima* because they have marginally winged seeds and persistent columellae extending most of the length of the locules.

Initially, the genus *Schima* was established in 1823, based on the type species *S. noronhae* Reinwardt ex Blume from Indonesia; the species has long pedicels, two caducous bracteoles, five suborbicular, ca.5 mm sepals, globose 5-loculed capsules and marginally winged seeds^19^. Since then, additional *Schima* species have been reported from China and adjacent areas. At present, it is generally acknowledged that there are a total of 20 species belonging to *Schima*, and 13 of them are distributed in China^3^. Compared with extant species of *Schima*, the new fossil species is distinguished by its longer pedicels, smaller sepals and larger globose fruits. Among extant *Schima* plants in China, the sepals are normally larger than 5 mm in diameter, and there are only two species (*S. argentea* and *S. superba*) with sepals measuring 2 mm in diam.; but they both have shorter pedicels (1–2 cm). Moreover, the size of the fruits is different. In fruit gross morphology *S. superba* can be viewed as the closest relative to the new fossil species.

To date, three fossil fruits of *Schima* have been reported, among which two species, *S. macrocalycalis* Mai and *S. lignitica* (Menzel) Mai, are from Germany^14^; another one, namely *S. nanlinensis* Li, Awasthi, Yang and Li., from China^15^. The fruit of *S. lignitica* is ovoid to oblong which can easily be distinguished from our fossils. The difference between the new species and *S. macrocalycalis* is that the latter has larger and suborbicular sepals. *S. nanlinensis* differs from the new fossil species in having depressed globose, and smaller capsules (0.8–1.1 cm). Detailed morphological comparison of the new species and similar species along within *Schima* is summarized in Table 1.

**Table 1:** Morphological comparison of *Schima kwangsiensis* with selected fossil and extant species^14, 15^.

	Pedicel length(cm)	Sepal shape	Sepal wide(mm)	Fruit shape	Fruit diameter(cm)	Seed shape	Seed Size(mm)	locality	age
*S. superba*	1–2	semiorbicular	2–3	subglobose	1–2	reniform	8–9 × 5–6	Southern China, Ryukyu Japan	Extant
*S. kwangsiensis*	3–5	semiorbicular	2–3	globose	2–2.5	reniform	7.5–8 × 4.5–5	Guangxi China	Late Oligocene
*S. nanlinensis*	—	—	Ca.2.	Depressed globose	0.6–0.9 × 0.8–1.1	reniform	6.5–7.5 × 4–5	Yunnan China	Miocene
*S. macrocalycalis*	—	Orbicular	—	subglobose	1.2–2.2	—	—	Saxony Germany	Eocene

### Systematic implication

Theaceae is a large and complex family in angiosperms that includes approximately 19 genera and 600 species^3, 6^. The classification of Theaceae has been disputed since the family was established. Traditionally, it is made up of two subfamilies Theoideae and Ternstroemioideae^5, 20–22^. However, a series of molecular studies suggested that the two subfamilies should be regarded as separate families^10, 23–25^. In APG III system of 2009, Ternstroemioideae was removed from the Theaceae to form the Ternstroemiaceae together with *Pentaphylax* (Pentaphylacaceae)^26^. Nevertheless, evolutionary trends within the Theaceae *s.s*. ( = Theoideae) remain controversial, which was shown by quite different classification systems^6, 27^. Specifically, the evolutionary significance of pedicel length has been subject to intense debate during the past few decades^6–8^. Keng (1980) even suggested that species with a long pedicel are more primitive than those with a short pedicel^28^, but the hypothesis is controversial due to the lack of definitive cladistic and fossil evidence. Hence, the mummified fossil fruits with intact pedicels described here could shed light on the early evolution of the genus *Schima*, and even of the family Theaceae.

**Figure 6:**
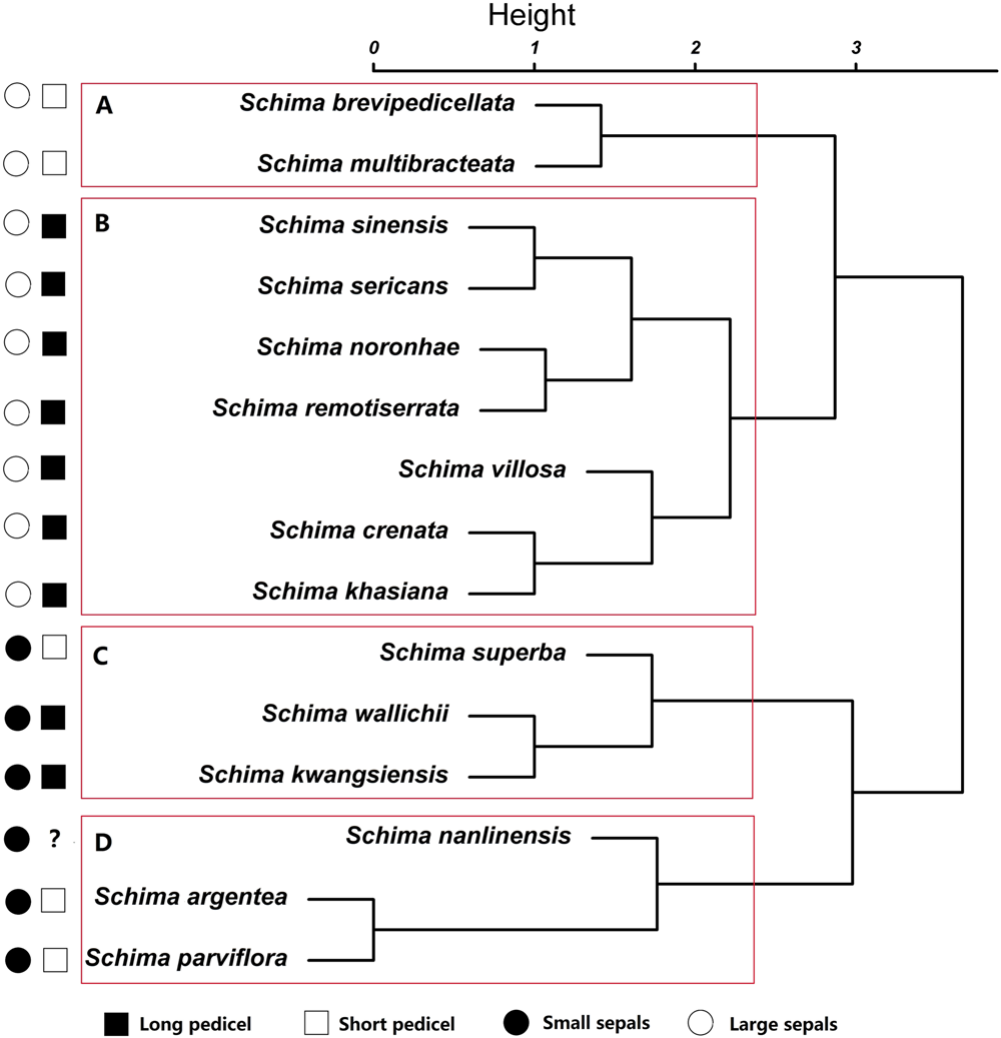
A cluster dendrogram of fossil and extant species of *Schima* in China based on fruit characters, generated and drawn by X. G. Shi using R (http://www.r-project.org/) and Adobe Photoshop CS5.

In order to gain a clearer taxonomic position of *Schima kwangsiensis* within the genus, a clustering analysis based on eight fruit characters (Tables 2, 3) for two fossil species and 13 extant species of the genus in China was performed using a modification of Ward’s method in R package^29^. The result shows that the species within *Schima*, including both the fossil and extant species, may be divided into four groups. As shown in the dendrogram (Fig. 6), clade A consists of only two species *S. brevipedicellata* and *S. multibracteata*, and is distinguished from the others by having short pedicels and large sepals. Clade B, represented by *S. sinensis, S. sericans, S. noronhae, S. remotiserrata, S. villosa, S. crenata*, and *S. khasiana*, mainly bears long pedicels and large sepals. Clade C, including the new fossil *S. kwangsiensis* and two extant species *S. superba*, *S. wallichii*, is characterized by having long pedicels and small sepals (there is one exception). The last clade, comprising one fossil species *S. nanlinensis*, and two extant species, *S. argentea* and *S. parviflora*, has small sepals and short pedicels. Furthermore, among 13 extant species, eight taxa have long pedicels and five taxa have short pedicels. It is obvious that the two morphological characters, pedicel and sepal size, are tightly correlated with each other in the genus *Schima* (Fig. 6). In general, the cluster analysis shows that the new fossil species is closely similar in pedicel and calyx size to two extant species (*S. wallichii* and *S. superba*) in the genus, implying that they may belong to an ancient taxon that predated the others.

**Table 2:** Characters and character states used for cluster analysis of fossil and extant *Schima* species.

1. Pedicel length: >2 cm (0); 1–2 cm (1);
2. Bracteole number: >2(0); 2 (1)
3. Bracteole position: away from sepal(0); close to sepal(1)
4. Sepal width: ≤3 mm (0); >3 mm (1)
5. Sepal shape: orbicular(0); semiorbicular(1)
6. Fruit shape: subglobose (0); depress globose(1)
7. Fruit diameter: >2 cm (0); ≤2 cm (1)
8. Seed size (length): >10 mm (0); ≤10 mm (1)

**Table 3:** Data matrix for character states of *Schima*.

species		characters							
		1	2	3	4	5	6	7	8
Extant species	*Schima villosa*	0	1	1	1	1	1	0	0
	*Schima noronhae*	0	1	0	1	1	0	0	0
	*Schima brevipedicellata*	1	1	?	1	1	1	0	1
	*Schima argentea*	1	1	0	0	1	0	1	1
	*Schima multibracteata*	1	0	?	1	1	?	?	?
	*Schima wallichii*	0	1	0	0	0	0	0	0
	*Schima sinensis*	0	1	1	1	1	0	1	0
	*Schima crenata*	0	1	1	1	0	0	0	1
	*Schima remotiserrata*	0	1	0	1	1	0	1	?
	*Schima superba*	1	1	1	0	0	0	0	0
	*Schima khasiana*	0	1	1	1	0	0	0	0
	*Schima sericans*	0	1	1	1	1	0	1	1
	*Schima parviflora*	1	1	0	0	1	0	1	?
Fossil species	*Schima nanlinensis*	?	?	?	0	?	1	1	1
	*Schima kwangsiensis*	0	1	0	0	0	0	0	1

### Paleogeography and Paleoecology

The origin and biogeographic history of *Schima* is little known because of the paucity of the fossil records. Up to now, only five definitive fossil occurrences have been reported in the world. Three fossil species are recognized on the basis of fruits, including *Schima macrocalycalis* from the middle Eocene of Germany, *Schima lignitica* from the late Miocene of Germany^14^, and *Schima nanlinensis* from the Miocene of Yunnan, China^15^. One wood fossil, *Schima protowallichii* occurs in the Miocene of Japan^30^, and a leaf fossil with well preserved cuticle was described as *Schima mataschensis*, is from the late Miocene of Styria, Austria^31^. In addition, one seed fossil, *Schima euryoides*once was reported from the late Eocene of Germany. However, its identity is doubtful as it has horseshoe-shaped embryos, a feature not found in *Schima* ^32^.

The *Schima* fruits reported here are the earliest fossils of this genus in Asia, and imply that this genus probably first appeared in Asia, its modern distribution area, by the late Oligocene. Moreover, the new fossil record is important because of the excellent preservation. Extant *Schima* has a small and lightweight seed with a marginal wing and the special structure of seeds aids long distance dispersal by wind^6^. The seeds of *Schima* fossils are morphologically similar to those of the extant genus implying that little change has occurred in the shape of seeds’ wing during the past 23 million years.

**Figure 7:**
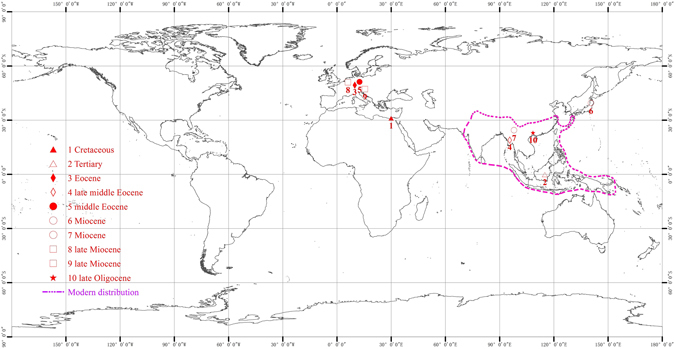
Map showing modern and fossil distribution of *Schima* and related fossil taxa, generated by X. G. Shi, using Arcgis 9.3 (ESRI, Redlands, CA, USA).

In addition to the fossils considered above, four species of *Schimoxylon*–a wood genus resembling *Schima* were reported, among which *Schimoxylon dachelense* was from the Upper Cretaceous of Egypt^33^, *Schimoxylon g ordonioides* was from the Tertiary deposits of Borneo of uncertain age^34^, *Schimoxylon altingioides* was from the Eocene of Germany^35^, and *Schimoxylon benderi* was from the late middle Eocene of Myanmar^36^ (Fig. 7). According to these fossil records, *Schima* possibly appeared by Late Cretaceous and achieved a widespread distribution from low-latitudes to middle latitudes during the Tertiary and was more widespread in the Northern Hemisphere than today. It suggests the ancient species of *Schima* probably originated in Northern Africa or Western Europe and further dispersed to the regions of Asia. However, the genus apparently experienced subsequent extinction both in Europe and in North Africa later and is now confined to subtropical and tropical zones within South China and Southeast Asia.

Extant *Schima superba*, which has similar fruits, is a tree up to ca. 30 m tall and occurs in evergreen broadleaved forests with a wide distribution in the subtropical and tropical montane areas at altitudes of 100–2500 m^37^. It is likely that *Schima kwangsiensis* may have been tall trees and grown in a similar environment. Additionally, preliminary investigations suggest that the mummified flora reported here mainly comprises Fagaceae, Theaceae, Bambusoideae, Anacardiaceae, Annonaceae, and Nyssaceae representing flowering plants, and Polyporaceae of the higher fungi^16^. These fossil taxa provide a glimpse of ancient ecological environments in Guangxi, indicating there has been a typical montane evergreen broad-leaved forest in southern China since at least the late Oligocene. The dominant species were mainly *Schima kwangsiensis* and some groups of Fagaceae in the tree layer, which grew under a warm moist forest environment.

## Methods

The fossil fruits were exceptionally well-preserved, and include intact pedicels and seeds. The specimens were thoroughly washed in water and dried in air. They were observed and photographed using a stereoscopic microscope (Zeiss Stereo Discovery V20). The seed micromorphology was investigated using a scanning electronic microscope (SEM Quanta 400 F). Next, the resulting images were processed with Adobe Photoshop CS5 (Adobe Inc., San Jose, California, USA). The extant fruits of *Schima superba* for comparison were collected from Heishiding Nature Reserve, Guangdong Province. To prevent the potential fracturing on drying, all the megafossil specimens used herein are preserved in a mixed solution of 50% alcohol and 100% glycerol with the volume ratio of 10:1 and deposited at the Museum of Biology of Sun Yat-sen University (SYS), Guangzhou, China. The terminology used to describe the fruit and seed follows Keng^8^, and Min & Bartholomew^3^.

Clustering analysis was performed using R (http://www.r-project.org/). The Excel file containing the binary data was imported to R package and the 0/1 matrix was used to calculate Euclidean distance. The resultant distance matrix was employed to construct dendrograms using hierarchical cluster analysis with Ward’s algorithm to infer genetic relationships^38^.
